# Impact of Residential Greenness on Preschool Children’s Emotional and Behavioral Problems

**DOI:** 10.3390/ijerph110706757

**Published:** 2014-06-27

**Authors:** Birute Balseviciene, Liuda Sinkariova, Regina Grazuleviciene, Sandra Andrusaityte, Inga Uzdanaviciute, Audrius Dedele, Mark J. Nieuwenhuijsen

**Affiliations:** 1 Department of Theoretical Psychology, Vytautas Magnus University, K. Donelaicio str. 58, Kaunas 44248, Lithuania; E-Mail: l.sinkariova@smf.vdu.lt; 2 Department of Environmental Sciences, Vytautas Magnus University, K. Donelaicio str. 58, Kaunas 44248, Lithuania; E-Mails: r.grazuleviciene@gmf.vdu.lt (R.G.); s.andrusaityte@gmf.vdu.lt (S.A.); i.uzdanaviciute@gmf.vdu.lt (I.U.); a.dedele@gmf.vdu.lt (A.D.); 3 Centre for Research in Environmental Epidemiology (CREAL), Barcelona, E-08003, Spain; E-Mail: mnieuwenhuijsen@creal.cat

**Keywords:** proximity to city parks, residential greenness, children’s emotional and behavioral problems, parenting stress

## Abstract

This study investigated the effects of the proximity to city parks and the influence of residential greenness on children’s emotional and behavioral problems. This cross-sectional study included 1,468 mothers of children (ages 4 to 6) who were residents of the city of Kaunas, Lithuania. The mothers and their children were enrolled in the FP7 PHENOTYPE project study. The mothers reported on their parenting stress and their children’s mental health. Residential greenness was characterized as an average of the satellite-derived normalized difference vegetation index (NDVI) in a 300 m buffer around each home address, and the proximity to city parks was defined as the distance from the subject’s residence to the nearest park. Linear regression models were used to investigate the association among the residence distances from city parks, greenness and children’s mental health problems. Farther residential distance from city parks was associated with worse mental health (except for the emotional problems subscale) in children whose mothers had a lower education level. More residential greenness was associated with worse mental health (more conditional problems and less prosocial behavior) in children whose mothers had a higher education level. These relationships have important implications for the prevention of emotional and behavioral problems in children.

## 1. Introduction

Children’s emotional and behavioral problems are currently an area of great concern. Approximately one-third of pre-school-aged children have behavioral or emotional difficulties [1,2]. A substantial amount of research has analyzed the causes and mechanisms of these problems [1,2,3,4,5,6,7,8]. Unfortunately, because of the high prevalence of children’s mental health problems [1,2], we assume that the current treatment of these problems and the intervention or prevention programs for such children are often not available or sufficiently effective. To improve this situation, traditional mental health risk assessment (in which risk factors are primarily included from only one main area) [1,2,3,4,5,6,7,8] must be expanded to incorporate and consider the combined exposure of a diverse array of environmental agents, such as exposure to the physical environment and social or family environmental factors [9].

Recent research has found that the main factors that impact emotional and behavioral problems are often influenced by individual and contextual factors [3,4,7,8,9]. According to developmental psychology, children’s age and gender are two of the most important individual factors [1,2,3,4,5,6,7,8]. The relevant contextual factors may include family socioeconomic status (SES), parent’s age and family situation, parenting stress and other various environmental exposures [1,2,3,4,5,6,7,8]. Among other factors, parenting stress has recently been subject to vigorous research in terms of its effects on child development [5,6,7,8]. Convincing evidence has linked parenting stress to reports of child behavioral and emotional problems, adjustment difficulties and internalizing or externalizing problems [5,7,8]. An additional contextual factor that may influence children’s mental health is the natural environment [10,11,12,13,14]. Numerous studies have shown strong associations between natural spaces and well-being in child and adult populations [10,11,12,13,14,15,16,17,18,19,20,21,22,23,24,25,26,27,28,29,30,31,32]. The research suggests that children may suffer long-term developmental consequences as a result of limited experiences in nature [10]. Moreover, environmental pollution affects both mental and physical health [33]. Studies have shown that contact with nature may not only decrease the symptoms of mental diseases but also positively affect children’s mental development [12]. Research has shown that exposure to green spaces, such as parks, trees, and gardens, has a positive influence on stress reduction, attention, and emotional state in children, young people, and adults [11,12,15,16,17,28,30]. Other research has focused on measures such as views through windows, access to gardens, and images of nature rather than direct contact with nature [17,18,23]. Additional studies have analyzed associations between nature and attention problems in children but have not studied other mental health problems [15,16,27,28,29]. In addition, several studies have analyzed the benefits of activities, especially outdoor physical activities, in primarily adult populations [21,23,25,26]. Recently, some epidemiological studies have examined the associations between nature and health at the population level [19,20,21]. These studies were conducted with the general population, primarily adults. The results showed that close proximity to nature had a positive impact on health [19,20]. These particular associations were stronger in small towns, in lower socioeconomic groups, and in youth and elderly people [14,19,20]. Some studies have noticed that availability of nature differs in socioecomic groups [12]. Maas *et al.* [19] indicated that the annual prevalence rates of depression and anxiety disorder disease clusters were lower in areas with more green spaces within a 1 km radius. These relationships were the strongest for children and for lower socioeconomic classes. Huynh *et al*. found that the relationship between more green space and reduced disease occurrence was stronger in slightly urban areas than in highly urban areas [14]. Sugiyama *et al*. noted that the perception of neighborhood greenness was associated with recreational walking and better physical and mental health [23]. In turn, physical activity is associated with better mental health [34]. Despite the above mentioned studies, there is a general lack of robust evidence of the link between green space and children’s mental health, which may be due to the inherent difficulties in quantifying non-physical health benefits, especially in children. The results of previous studies are important but appear to differ in terms of the significance and strength of the relationships [12,21,30]. It seems that nature, specifically parks, can provide various benefits to children and their mental health. It is important to analyze how nature affects children, especially because children are more vulnerable to the negative and positive effects of the environment than are adults [35]. This difference in impact occurs because of fundamental differences in children’s physiology, metabolism, absorption and exposure patterns [12]. Furthermore, whereas adults may decide where, when and how to interact with natural spaces, children do not have the same independent mobility because their parents’ decisions may influence where they go and where they play. At times, children’s decreased contact with nature is not solely due to their interest in playing indoors (for example, playing computer games). Instead, their parents may be concerned about their safety and thus do not allow them to play outdoors. As mentioned above, cross-sectional studies have suggested positive relationships between green space and health; however, the identification of the causal pathway can be a complex process. Thus, it is crucial to define the term “nature”. Previous studies have provided different definitions of the term. For example, the definitions “number of green spaces in a neighborhood”, “proximity to green spaces” and “the greenness of natural spaces” differ. Moreover, subjective and objective measures of green spaces vary. For example, de Vries *et al.* measured the number of green spaces and proximity to green spaces [32], Sugiyama *et al*. [23] analyzed the perceived neighborhood greenness, whereas Huynh *et al.* [14] and Maas *et al.* [19] only analyzed proximity to such spaces. Kuo *et al.* [27], Taylor and Kuo [28], Wells [17], and Wells *et al.* [18] examined the greenness of a neighborhood, which was measured according to parents’ perceptions of the naturalness of their home and their children’s play setting.

Most of the above studies were conducted in countries whose seasonal climate changes are not as extreme as those in Lithuania. Additionally, studies have shown that spending time in parks is associated with attitudes toward nature, which are culture-specific [14]. Therefore, it may be assumed that the results of studies that were conducted in countries with different climate conditions, different numbers of parks and green spaces and even different cultural attitudes may not be generalizable to Baltic countries or other countries in the region. 

Most of the above mentioned studies were epidemiological [19,20], and only a few of them considered the psychological factors of mental health in children [15,16,17,18,27]. Most of them included some questions about children’s mental health [16,19,20,27], but few applied certified instruments to assess children’s mental health [15,17,18]. Moreover, most of the studies failed to consider psychological factors, such as parenting stress [15,16,17,18,19,20,27]. However, as mentioned above, parenting stress is one of the most important contextual factors that are associated with children’s mental health [5,6,7,8]. 

The current study was conducted as part of the Positive Health Effects of the Natural Outdoor Environment in Typical Populations in Different Regions in Europe (PHENOTYPE) project, which was funded by the European Commission Seventh Framework Program [36]. 

To our knowledge, the current study is one of the first to use a large sample of child and mother pairs. This study employed psychologically valid instruments to measure the mental health of children and their mothers. Additionally, proximity to city parks and residential greenness were measured quantitatively and objectively. In this paper, we hypothesized that the impact of green space on mental health problems depends on the proximity of the place of residence to city parks and the greenness of the residential place. 

## 2. Methods

### 2.1. Study Population

This study used survey data on women who were recruited to a pregnant women’s cohort study from 2007–2009. This study is a multi-wave cohort study that was conducted in the city of Kaunas, Lithuania. Briefly, the data from three follow-ups have been collected and are currently available to researchers. The first wave was completed during the women’s first trimester of pregnancy. At enrollment, the women reported their age, socioeconomic and demographic measures, smoking preferences (non-smokers or smoke at least one cigarette per day), body mass index and other variables. The second wave was completed after childbirth and included data on the women’s residential history, job during pregnancy, health behavior and other variables. The individual-level SES predictors were education level and type of occupation. The third wave of interviews was conducted in 2013. In the current paper, we analyzed the data from this third wave of 1468 mothers of 4- to 6-year-old children residing in the city of Kaunas, Lithuania. The study was approved by the Lithuanian Bioethics Committee, and parental informed consent was obtained from all of the participants. The mothers’ questionnaire responses were used to categorize the demographic measures, the children’s emotional and behavioral problems, and parenting stress. 

### 2.2. Measures

#### 2.2.1. Parenting Stress

The Simplified Version of Parenting Stress Index-Short Form (S-PSI/SF; Abidin [5], Haskett *et al*. [6], Yeh *et al*. [37]) is used for the early identification of dysfunctional parent-child interactions, parenting stress and difficulties with a child. The S-PSI/SF consists of a 15-item test. (It yields four scores, including a child domain score, a parent domain score, a child-parent interaction score and a total stress score. In the current study, the total stress score was used. An example item is “I find myself giving up more to meet my children’s needs”. The participants’ responses are provided on a 5-point Likert scale that ranges from 1 (indicating strong agreement) to 5 (indicating strong disagreement). Higher scores indicate greater parenting stress. The S-PSI/SF demonstrated good factorial and construct validity and internal reliability in groups of Chinese women and Lithuanian women [37,38]. The internal consistency of the S-PSI/SF, as measured by Cronbach’s alpha, was 0.905. 

#### 2.2.2. Children’s Mental Health Problems (Lithuanian Version (SDQ))

The Strengths and Difficulties Questionnaire (SDQ; Gintiliene *et al*. [39]) is a brief behavioral screening questionnaire that was designed to assess prosocial behavior and emotional and behavioral problems in children on 5 scales. In the Lithuanian sample study [40], the following five scales were used: conduct problems (e.g., “Often fights with other children and bullies them”); hyperactivity (e.g., “Constantly fidgeting or squirming”); emotional problems (e.g., “Many worries, often seems worried); peer relationship problems (e.g., “Gets along better with adults than with other children”); and prosocial behavior (e.g., “Considerate of other’s people feelings”). There are 5 items per subscale. All of the subscales except the prosocial behavior subscale are summed to generate a total difficulties score. The participants’ responses are provided on a 3-point Likert scale that ranges from 0 (indicating strong disagreement) to 2 (indicating strong agreement). Higher scores on the SDQ indicate more difficulties with emotional and behavioral problems, except for the prosocial behavior score, in which higher scores indicate improvements*.* The internal consistency of the SDQ total score in this study, as measured by Cronbach’s alpha, was 0.778 (compared to Gintiliene *et al*. [39]—0.790). The internal consistency of each subscale was as follows: conduct problems: 0.616, hyperactivity: 0.636, emotional problems: 0.633, peer relationship problems: 0.623 and prosocial behavior: 0.678. 

#### 2.2.3. Proximity to City Parks and Residential Greenness

All of the Kaunas city parks that were larger than 1 ha and generally had over 65% of their land covered with trees were regarded as city parks. The spatial land cover data sets for the city of Kaunas were obtained from the municipality. Small-scale green spaces, such as street trees, green roadsides or plain forests with no infrastructure, and agricultural green places and natural conservation areas were not included in the data set. In the current study, some Kaunas city forests that were not considered as city parks but had their own infrastructure were referred to as city parks. We estimated the distance from the participants’ current residential addresses to the nearest park using ArcGIS 10 software. When a home address was not available, the children’s kindergarten address was used. The proximity to a city park was not normally distributed. Therefore, for further analyses, this variable was transformed using the square root function.

The assessment of residential greenness was based on a normalized difference vegetation index (NDVI) that was derived from Landsat 7 Enhanced Thematic Mapper Plus (ETM+) data at 30 m × 30 m resolution. The NDVI is an indicator of greenness that is based on land surface reflection of visible (red) and near-infrared parts of the spectrum [40]. It ranges between −1 and 1, with higher numbers indicating more greenness. To achieve a maximum exposure contrast, we searched for available cloud-free Landsat ETM+ images from the summer. Based on this search, we generated a NDVI map using the image that was obtained on 27 June 2013. Surrounding greenness was defined as the average of the NDVI in a buffer of 300 m around the home address of each study participant; this variable was geocoded according to the address at the time of the questionnaire administration.

### 2.3. Statistical Analysis

We applied standard, non-hierarchical linear regression models to investigate the relationships among the proximity to city parks, residential greenness and children’s emotional and behavioral problems. Descriptive statistics were used to describe the main sociodemographic variables. All of the statistical analyses were performed using SPSS version 18.0 (SPSS Inc. Released 2009; PASW Statistics for Windows, Version 18.0., Chicago, IL, USA).

## 3. Results

### 3.1. Characteristics of the Study Population

Table 1 shows the definitions and descriptive characteristics of all of the variables that were used in the analyses. The participants included 1468 mothers and their 4- to 6-year-old children (49.3% were boys and 50.7% were girls). The mothers’ average age was 34 years (SD = 5.049, range 21–50 years), and the children’s ages varied as follows: 51.2% were 4 years old, 31.1% were 5 years old, and 17.7% were 6 years old. The participants were residents of the city of Kaunas, Lithuania. A total of 86.9% of the women lived with the father of the child. Additionally, 72.9% of the women were employed at the current time, and 94.9% of the children attended kindergarten.

**Table 1:** Characteristics of the study population.

Age (Years, Mothers)	33.75 (SD: 5.049, Range: 21–50)
Age (years, children)	
4 years	51.2%
5 years	31.1%
6 years	17.7%
Child’s gender	
Male	49.3%
Female	50.7%
Level of education	
Higher	79.80%
Lower	20.20%
Employment status	
Employed	72.9%
Unemployed	37.1%
Proximity to city parks (meters) Transformed proximity to city parks (meters)	666.97 (SD: 544.479; range: 0–3085) 23.89 (SD: 9.868; range: 1–58)

We divided the mothers’ education into the following two categories: lower education (primary school and secondary school) and higher education (college education and university degree). As shown in Table 1, there was considerable diversity in maternal education. Specifically, 79.8% of the mothers had a higher education level, and 20.2% of the women had a lower education level.

First, we analyzed the differences between the proximity to city parks and residential greenness, which differed according to the mother’s education. Whereas there were no differences in residential greenness according to the mothers’ education, it appeared that mothers with higher education levels lived farther from city parks than mothers with lower education levels (transformed proximity of 24.31 and 22.24, respectively, *p* = 0.0001). Because of these primary differences in the data, we analyzed all of the associations separately for the lower and higher education groups.

### 3.2. Associations between Children’s Mental Health Problems and Greenness

The main purpose of the current analysis was to assess the impact of proximity to city parks, residential greenness, mother and child sociodemographic factors and parenting stress on the children’s mental health problems. Linear, non-hierarchical regression analyses were conducted with each of the SDQ subscales and the total difficulties scale because the scales assessed different aspects of children’s mental health and had different associations with the other analysis variables. The independent variables included proximity to parks, residential greenness, parenting stress, child’s gender and child’s age; the dependent variable was the children’s mental health problems, as measured by the SDQ. All of the regression models were statistically significant (*p* = 0.0001).

In all of the models, parenting stress had significant impacts on the children’s mental health problems (Table 2). The child’s age was associated with the total difficulties, peer problems, and prosocial behavior subscales in the lower maternal education group. In the higher maternal education group, the child’s age was associated with the conditional problems subscale. The child’s gender was associated with all of the SDQ subscales except for emotional problems in the higher maternal education group. Based on further analyses, the proximity to city parks, the greenness of residential neighborhoods and their impacts on the children’s mental health are discussed in greater detail below.

The first regression model tested the effects of all of the predictors on the children’s mental health problems. In this model, the children’s mental health problems were assessed based on the SDQ total difficulties scale (Table 2). The proportions of variance that were explained by the models were moderate (the R scores in the lower education and higher education groups were 44.4% and 44.8%, respectively). The proximity to city parks had an impact on the children’s mental health problems (total difficulties subscale) only in the lower maternal education group. However, there was a statistically non-significant tendency of associations between residential greenness and children’s mental health in the higher maternal education group.

The second regression model tested the effects of all of the predictors on the children’s peer problems. According to the R coefficients, the proportions of variance that were explained by the models were 31% and 26% in the lower and higher maternal education groups, respectively. The proximity to city parks had an impact on the children’s peer problems in the lower maternal education group. As shown in Table 2, more difficulties occurred in children who lived farther from parks. Residential greenness did not significantly impact children’s peer problems in either the lower maternal education group or the higher maternal education group.

**Table 2:** Regression analysis of transformed proximity to city parks based on children’s residential address (TPC) and average of the NDVI in a 300 m buffer around each child’s residential address (NDVI) (other predictors: parenting stress ^1^, child’s sex ^2^ and child’s age ^3^).

SDQ Subscale	Predictors	Lower Education Group *N* = 296				Higher Education Group *N* = 1172			
		β Coefficients		R of Model		β Coefficients		R of Model	
**Total difficulties**	TPC	0.069	*	0.444	**	−0.012		0.448	**
	NDVI	1.293				2.286	#		
**Peer problems**	TPC	0.023	*	0.312	**	−0.003		0.262	**
	NDVI	0.238				0.481			
**Conditional problems**	TPC	0.026	*	0.331	**	−0.001		0.343	**
	NDVI	1.522	#			0.901	*		
**Emotional problems**	TPC	−0.002		0.381	**	−0.008	#	0.368	**
	NDVI	0.200				0.049			
**Hyperactivity**	TPC	0.026	*	0.258	**	−0.002		0.292	
	NDVI	−0.743				1.031	#		
**Prosocial behavior**	TPC	−0.029	*	0.243	**	−0.002		0.250	**
	NDVI	−0.197				−1.104	*		

Note: * < 0.05 ** < 0.01 # < 0.1; ^1^ In all models, higher parenting stress was associated with more mental health problems in children. ^2^ In the higher maternal education group, boys displayed more problems in the total difficulties, peer problems, conditional problems, and hyperactivity subscales, and girls demonstrated more prosocial behavior. ^3^ In the lower maternal education group, older children displayed more problems in the total difficulties and peer problems subscales. Older children also displayed more prosocial behavior and more emotional problems in both education groups. Younger children had more conditional problems in the higher maternal education group.

The third regression model tested the effects of all of the predictors on the children’s conditional problems. The proportions of variance that were explained by the models were rather high in both groups (the R scores in the lower maternal education group and higher maternal education group were 33.1% and 34.3%, respectively). In the lower maternal education group, more difficulties were reported for those children who lived farther from parks. Residential greenness was associated with children’s problems in the higher maternal education group (greener parks were associated with more problems), whereas a statistically non-significant tendency of these associations was found in the lower maternal education group.

The fourth regression model tested the effects of all of the predictors on the children’s emotional problems. The proportions of variance that were explained by the models were moderate (the R scores in the lower maternal education and higher maternal education groups were 38.1% and 36.8%, respectively). Neither proximity to city parks nor residential greenness was a significant predictor of children’s emotional problems in either of the education groups. However, we noted a statistically non-significant tendency of associations between proximity to city parks and children’s emotional health in the higher maternal education group.

The fifth regression model tested the effects of all of the predictors on the children’s hyperactivity. The model explained 25.8% and 29.2% of the hyperactivity problems in the lower and higher maternal education groups, respectively. The proximity to city parks had an impact on the children’s hyperactivity in the lower maternal education group. Hyperactivity increased as the children became farther from city parks. However, we observed a statistically non-significant tendency of associations between residential greenness and hyperactivity in the higher maternal education group.

The last regression model tested the effects of all of the predictors on the children’s prosocial behavior. The models explained 24.3% of the variance in the children’s prosocial behavior in the lower maternal education group and 25% in the higher maternal education group. The proximity to city parks had an impact on the children’s prosocial behavior in the lower maternal education group. Prosocial behavior increased when the children lived near city parks. The residential greenness was associated with prosocial behavior in the higher maternal education group (the children in areas with less residential greenness displayed more prosocial behavior).

## 4. Discussion

The aim of the present study was to analyze the associations among the proximity to city parks, residential greenness and children’s behavioral and emotional problems. As a number of authors previously reported, nature not only positively affects mental health but also serves as a buffer from diseases and helps to decrease stress [10,12,13,14,15,17,18,19,20,21,23,24,25,26,27,28,29,30,31,32,33,34,35]. The current study found a significant positive relationship between the children’s mental health problems (total difficulties, hyperactivity, peer and conditional problems) and proximity to city parks in the lower maternal education group. There is a lack of studies on the preschool children population, but the current results correspond with the findings of previous studies on other age groups. Maas *et al.* [19] and Huynh *et al.* [14] showed that proximity to green spaces impacted mental health and that the relationships between these two variables were stronger in lower socioeconomic groups. Additionally, these relationships were stronger for younger people [19], although the authors did not distinguish preschool children. In the current study, the proximity to city parks was a significant predictor of prosocial behavior in the lower maternal education group. It seems that green spaces, especially those with infrastructures (as in this study), encourage inhabitants to use them and help to create more activities that promote social interactions and simultaneously increase prosocial behavior [21,31,32]. It is possible that we found significant relationships between mental health problems and proximity to city parks only in the lower maternal education group because these mothers and their children live in worse conditions and, thus, are more affected by other stressors and are more vulnerable to negative and positive environmental exposures [14]. Furthermore, families with less education may have fewer possibilities to travel farther from their residences and, thus, typically remain close to their homes. This possibility may explain why we and other authors obtained significant or strong relationships in this sample but not in the higher maternal education sample [14,19].

Only one of the child mental health problems—emotional problems—was not predicted by the proximity to city parks in either of the maternal education groups, and the results from previous studies are contradictory. Some studies have confirmed these associations, but others have reported no or very weak associations [14,21,31]. The current study results did not confirm the significance of these associations. The previous studies that found such associations were conducted primarily in adult populations [31]. In the studies that were conducted in youth populations, these associations were weak, similar to the current study findings [14]. Of note, no previous studies examined preschool-aged children. We can assume that such controversial results emerge from different assessments of emotional problems and different age groups. In the previously mentioned studies, different aspects of emotional health associations with nature were analyzed, primarily in adult populations. Some studies estimated emotional health problems (with some children reaching clinical levels), whereas other studies analyzed emotional well-being. The current study assessed children’s emotional problems but not their emotional well-being. Most of the previous studies used one question to evaluate emotional health. In the current study, the mothers assessed their children’s emotional problems using a valid and reliable psychological instrument.

In the current study, we used a residential greenness index as one of the predictors of children’s mental health problems. As Sugiyama *et al*. [23], Wells [17], and Wells *et al*. [18] suggested, nearby nature is significant to an individual’s health, and its accessibility and greenness play an important role in mental health outcomes [15,16,17,18,23]. Given the lack of studies on preschool children’s mental health problems, there is limited evidence regarding their cognitive functioning and stress reduction in association with greenness. The parental-perceived or investigator-assessed naturalness of views through home windows or play in natural settings have been associated with better cognitive functioning [17], increased self-discipline [16], and milder symptoms of impulsivity in children with ADHD [15] and in children without ADHD [28]. All of these studies examined school-aged children. Wells *et al*. [18] indicated that perceived greenness was associated with cognitive functioning and served as a buffer from stressful life events for 3rd–5th grade children. The current study found that more residential greenness was associated with more conditional problems and less prosocial behavior in the higher maternal education group, but there were no significant associations between hyperactivity and residential greenness. In previous studies, the greenness or naturalness of natural spaces was measured according to the subjective opinions of parents [15,16,17,18,23,27], whereas the current study measured it objectively. We can assume that people with a higher education live in the suburbs (because living in the suburbs is more expensive than residing in cities). However, residence in the suburbs, especially in very green places, does not necessarily indicate longer time spent outdoors. Families may be concerned about their children’s safety and, thus, prevent them from playing outdoors. Moreover, Kaunas is a very green city, and there were no extreme differences in the greenness index between residential places. Finally, previous studies primarily analyzed the mental health problems of school-aged children, whereas the current study examined preschool children.

One of the strengths of this study was the analysis of parenting stress as a predictor of children’s mental health problems. Parenting stress is common among parents whose children have mental health difficulties [7,8,10]. Parenting stress also has a large effect on children’s mental health and is associated with the mothers’ mental health. It may also depend on green spaces and the greenness of the surrounding neighborhood. It can be assumed that the proximity to city parks and other green spaces not only reduces the mother’s stress and improves her well–being but also has indirect impacts on the children’s mental health. It is possible that mothers with better mental health display better parenting behaviors, even when the child has mental health problems. This hypothesis was partly confirmed by Kuo and Sullivan [29]. The researchers noted that individuals who lived close to nature were less aggressive toward their families. Moreover, children who live near green places, as various study results have shown, often show fewer or milder mental health problems. Consequently, the probability of parenting stress decreases. The current study employed a clear definition of the type of nature that was assessed. The proximity to city parks and the neighborhood greenness were assessed objectively, and the sample was rather large. However, the proximity to city parks was assessed as a straight line from the mother’s residential address to the city park without an analysis of direct accessibility, which is a limitation of this study. The current study also did not estimate park use and time spent in the park or in green places near the home. Future studies are needed to estimate the impact of the quality of greenery on park usage, time spent in the natural environment and children’s mental health.

Previously published data have shown that mental health problems that emerge in early childhood are risk factors for mental health problems in adolescence and adulthood [3,4]. Children’s emotional and behavioral problems should be treated as high risk factors for serious mental health problems that could be controlled by population-based interventions. Moreover, prevention and intervention programs are quite expensive and difficult to access and require highly educated specialists, whereas encouraging people to spend time in and live nearby nature is a more simple method. Therefore, to establish public policy for the prevention of children’s behavioral and emotional problems, future environmental epidemiological studies should consider the contribution of both individual and environmental factors on the study subjects.

## 5. Conclusions

The mental health of children of lower educated mothers is associated with residential proximity to city parks, whereas the prosocial behavior of children of higher educated mothers is associated with residential greenness. The findings of the present study extend the knowledge base about children’s mental health problems through the use of objective measures of proximity to city parks and residential greenness. This study was the first to control for parenting stress as one of the most powerful family predictors of children’s mental health problems. These results have an important public health application in supporting green urban planning policy and preventing children’s mental health problems. Future studies are required to explore the effects of natural spaces and exposure-response modifying factors on the development of children’s mental health problems in order to identify the underlying mechanisms.
